# Effects of acquisition device, sampling rate, and record length on kinocardiography during position-induced haemodynamic changes

**DOI:** 10.1186/s12938-020-00837-5

**Published:** 2021-01-06

**Authors:** Amin Hossein, Jérémy Rabineau, Damien Gorlier, Farhana Pinki, Philippe van de Borne, Antoine Nonclercq, Pierre-François Migeotte

**Affiliations:** 1 LPHYS, Université Libre de Bruxelles, Brussels, Belgium; 2 Department of Cardiology, Erasme Hospital, Université Libre de Bruxelles, Brussels, Belgium; 3 BEAMS, Université Libre de Bruxelles, Brussels, Belgium

**Keywords:** Cardiac contractility, Cardiac kinetic energy, Cardiac strength, Ballistocardiography, Seismocardiography, Kinocardiography, Wearable monitoring, Head down tilt, Head up tilt

## Abstract

**Background:**

Kinocardiography (KCG) is a promising new technique used to monitor cardiac mechanical function remotely. KCG is based on ballistocardiography (BCG) and seismocardiography (SCG), and measures 12 degrees-of-freedom (DOF) of body motion produced by myocardial contraction and blood flow through the cardiac chambers and major vessels.

**Results:**

The integral of kinetic energy ($$ iK$$) obtained from the linear and rotational SCG/BCG signals was computed over each dimension over the cardiac cycle, and used as a marker of cardiac mechanical function. We tested the hypotheses that KCG metrics can be acquired using different sensors, and at 50 Hz. We also tested the effect of record length on the ensemble average on which the metrics were computed. Twelve healthy males were tested in the supine, head-down tilt, and head-up tilt positions to expand the haemodynamic states on which the validation was performed.

**Conclusions:**

KCG metrics computed on 50 Hz and 1 kHz SCG/BCG signals were very similar. Most of the metrics were highly similar when computed on different sensors, and with less than 5% of error when computed on record length longer than 60 s. These results suggest that KCG may be a robust and non-invasive method to monitor cardiac inotropic activity.

*Trial registration* Clinicaltrials.gov, NCT03107351. Registered 11 April 2017, https://clinicaltrials.gov/ct2/show/NCT03107351?term=NCT03107351&draw=2&rank=1.

## Background

Cardiac contractions occur more than 2 billion times during an average lifespan. Early detection of any cardiac impairment would allow for intervention and the prevention of irreversible damage [1]. Therefore, the cardiac chronotropic and inotropic states of patients at risk of a cardiovascular complication should be measured regularly. Currently, the main non-invasive modes of cardiac function assessment include electrocardiography (ECG), echocardiography (Echo), and cardiac magnetic resonance (cMR). The gold standard for cardiac contractility assessment is cMR. However, Echo is the most commonly used technique in daily clinical practice. Both cMR and Echo require trained operators, and are time-consuming. This makes them unsuitable for regular, weekly, or monthly assessments for all at-risk patients. Alternative technologies are therefore needed for more regular and autonomous measures of cardiac function.

Recently, there has been renewed interest in ballistocardiography (BCG) and seismocardiography (SCG) for automated assessment of the inotropic state of the heart [2]. These technologies are based on measurements of body movements induced by cardiac contraction, and blood flow in the cardiac chambers and major vessels of the heart [3]. While BCG primarily measures overall body recoil in response to blood ejection from the heart, and pulsatile blood flow in the aorta and its main branches [3], SCG primarily records precordial accelerations or vibrations of the chest that result from the complex series of movements caused by myocardial contraction [4]. The relationship between cardiac contractility and ballistic signals was identified, and extensively studied by Starr in the twentieth century [3]. Several studies have shown that the signals recorded with BCG and SCG are good indicators of myocardial function and dysfunction [5-7]. A review of recent advances in the field SCG was published by Taebi et al. recently [8].

We previously reported on kinocardiography (KCG) as a subject-specific calibrated combination of linear and rotational SCG and BCG techniques. KCG measures a total of 12 degrees-of-freedom (DOF) of body motion. This consists of 6-DOF from three-dimensional (3D) linear and 3D angular motion recorded from sensors attached to the sternum (SCG) and 6-DOF (3D linear and 3D angular) from whole-body motion recorded from the lower back area (BCG). We introduced the time integral of kinetic energy (iK) over the cardiac cycle as a measure of total cardiac mechanical effort [6]. We showed that, in healthy individuals, this metric could detect dobutamine-induced haemodynamic changes with a high degree of accuracy, sensitivity, and specificity (≥ 94%). Furthermore, the metric iK, observed with increasing doses of dobutamine injection, was correlated with stroke volume (SV) and cardiac output (CO) [6]. The same iK metric showed a significant increase during a brief end expiratory breath hold, and an even larger increase during subsequent spontaneous inspiration, possibly due to its sensitivity to changes in the right heart volume [9]. Previous studies have also shown that cardiovascular deconditioning resulting from long-duration head-down tilt bed rest was visible on KCG metrics. In particular, the evolution of linear BCG iK (${{iK}}_{\text{Lin}}$) followed changes in stroke volume [10, 11]. KCG metrics show promising results, but would benefit from further validations. Indeed, to date, there have been no studies on inter-sensor acquisition differences. Furthermore, in our previous study [6], KCG metrics were computed on SCG and BCG signals acquired at 50 Hz, while these signals are acquired at higher frequencies which range from 100 Hz to 1 kHz [2, 12].

The BCG and SCG signals are prone to artefacts from non-cardiac voluntary or involuntary movements of the body due to their sensitivity [2, 3]. The ensemble average (EA) technique minimises artefacts by pooling and temporally aligning the signals from several heart beats to construct a new signal that represents an average cardiac cycle [8]. In a linear and a stationary system, the longer the record, the better the EA would represent this average heartbeat [13]. However, it is impractical to record signals during a very long period. To our knowledge, there are no studies on the quantification of the effects of record duration on the EA constructed on SCG and BCG signals, and consequently on the KCG metrics.

The acute head-down tilt (HDT) and head-up tilt (HUT) positions are known to increase [14] and decrease [15] the SV, respectively. While the effects of postural changes on BCG and SCG have been reported [16, 17], there are no studies on the effect of acute HDT and HUT on BCG and SCG. In this study, healthy volunteers were tested in a tilt test protocol at baseline, and after HDT and HUT, used to widen the haemodynamic states on which the validation was performed, and to observe the effects of these on the KCG metrics.

This study aimed to consolidate the technical aspects of KCG by: (1) comparing KCG metrics acquired using two different devices; (2) validating that a sampling rate of 50 Hz allows a precise measure of KCG-derived metrics; (3) analysing the effects of the EA window length on these KCG metrics; and (4) documenting the effects of acute changes in body position on the KCG parameters, and comparing them to existing literature.

## Results

The cohort HR and KCG parameters in the supine, HDT, and HUT positions, are presented in Table 1.

**Table 1:** Physiological and KCG parameters presented as median [Q1; Q3] during supine (baseline) and for each position (HDT, and HUT)

Measuring device	Parameter		Supine	HDT	HUT
ECG-Dev1	HR (bpm)		65.3 [56.4; 75.3]	64.7 [57.2; 72.9]	78.5 [71.7; 81.1]^†^
Dev1	SCG	${{{iK}}_{{{\text{Lin}}}}}$ (µJ s)	66.5 [40.3; 99.9]	77.6 [50.2; 139]	33.6 [24.3; 46.4]^†^
		${{{iK}}_{{{\text{Rot}}}}} { }$ (µJ s)	331 [266; 389]	270 [189; 533]	236 [124; 355]*
	BCG	${{{iK}}_{{{\text{Lin}}}}} { }$ (µJ s)	1.3 [0.7; 1.9]	1.8 [1.1; 3.8]^†^	0.7 [0.6; 1.9]
		${{{iK}}_{{{\text{Rot}}}}} { }$ (µJ s)	2.7 [1.9; 4.3]	7.8 [3.7; 14.7]*	3.3 [1.5; 11.6]
Dev2	SCG	${{{iK}}_{{{\text{Lin}}}}} { }$ (µJ s)	61 [37.3; 91.2]	70 [44.2; 124]	27 [19.6; 42.4]^†^
		${{{iK}}_{{{\text{Rot}}}}} { }$ (µJ s)	335.6 [268; 410]	273.5 [202; 551]	257.4 [128; 372]*
	BCG	${{{iK}}_{{{\text{Lin}}}}} { }$ (µJ s)	1.4 [0.7; 2.0]	1.9 [1.1; 3.7]^†^	0.7 [0.6; 1.8]
		${{{iK}}_{{{\text{Rot}}}}} { }$ (µJ s)	4.7 [3.6; 7.3]	11.7 [8.0; 16.4]*	5.7 [3.4; 11.9]

Paired comparison for each parameter to compare HDT and HUT to Supine. *p < 0.005; ^†^*p* < 0.0001

### Sampling rate validation

**Fig. 1:**
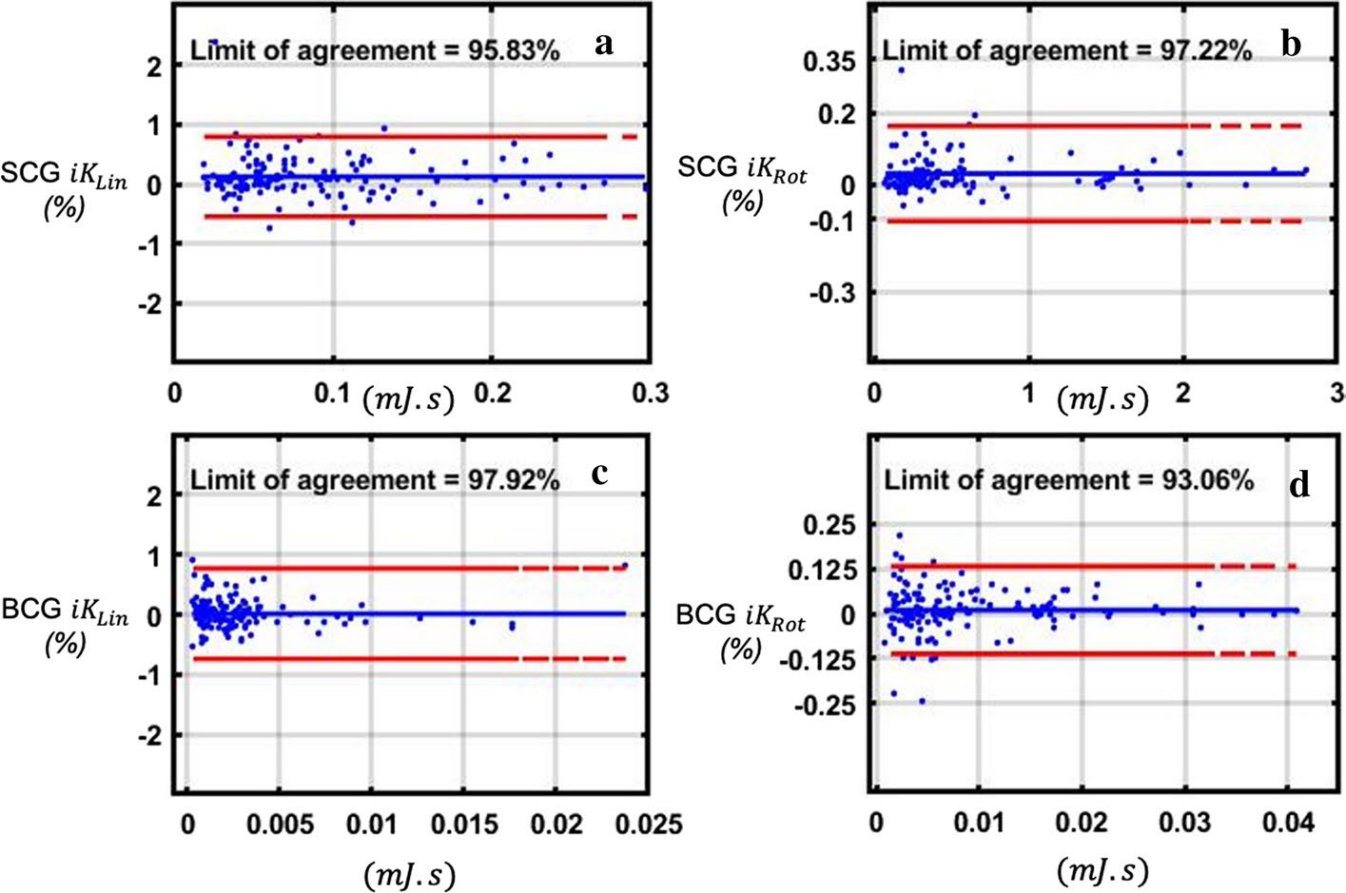
Bland–Altman Plot of the KCG metrics computed from Dev1 acquisitions at 1 kHz versus 50 Hz. The differences (Dev1 1 kHz–Dev1 50 Hz) are expressed as a percentage of the value on the axis, i.e., proportionally to the magnitude of measurements, are plotted as a function of the mean value of the two measures. **a** SCG ${{{iK}}_{{{\text{Lin}}}}}$; **b** SCG ${{{iK}}_{{{\text{Rot}}}}}$; **c** BCG ${{{iK}}_{{{\text{Lin}}}}}$; **d** BCG ${{{iK}}_{{{\text{Rot}}}}}$

Bland–Altman plots were generated for each metric computed with Dev1 on the initial 1 kHz acquisition against its equivalent on the down-sampled version at 50 Hz. The difference between the two paired measurements was plotted against the mean of the two measurements. No trends were observed for any of the computed metrics (Fig. 1). The limits of agreement of the compared data were 97.92% for BCG ${{iK}}_{\mathrm{Lin}}$, 93.06% for BCG ${{iK}}_{\mathrm{Rot}}$, 95.83% for SCG ${{iK}}_{\mathrm{Lin}}$, and 97.22% for SCG ${{iK}}_{\mathrm{Rot}}$. The mean difference in percentage between the two methods for each metric was 0.015%, 0.01%, 0.1%, and 0.03%, respectively (Fig. 1).

### Sensors comparison

**Fig. 2:**
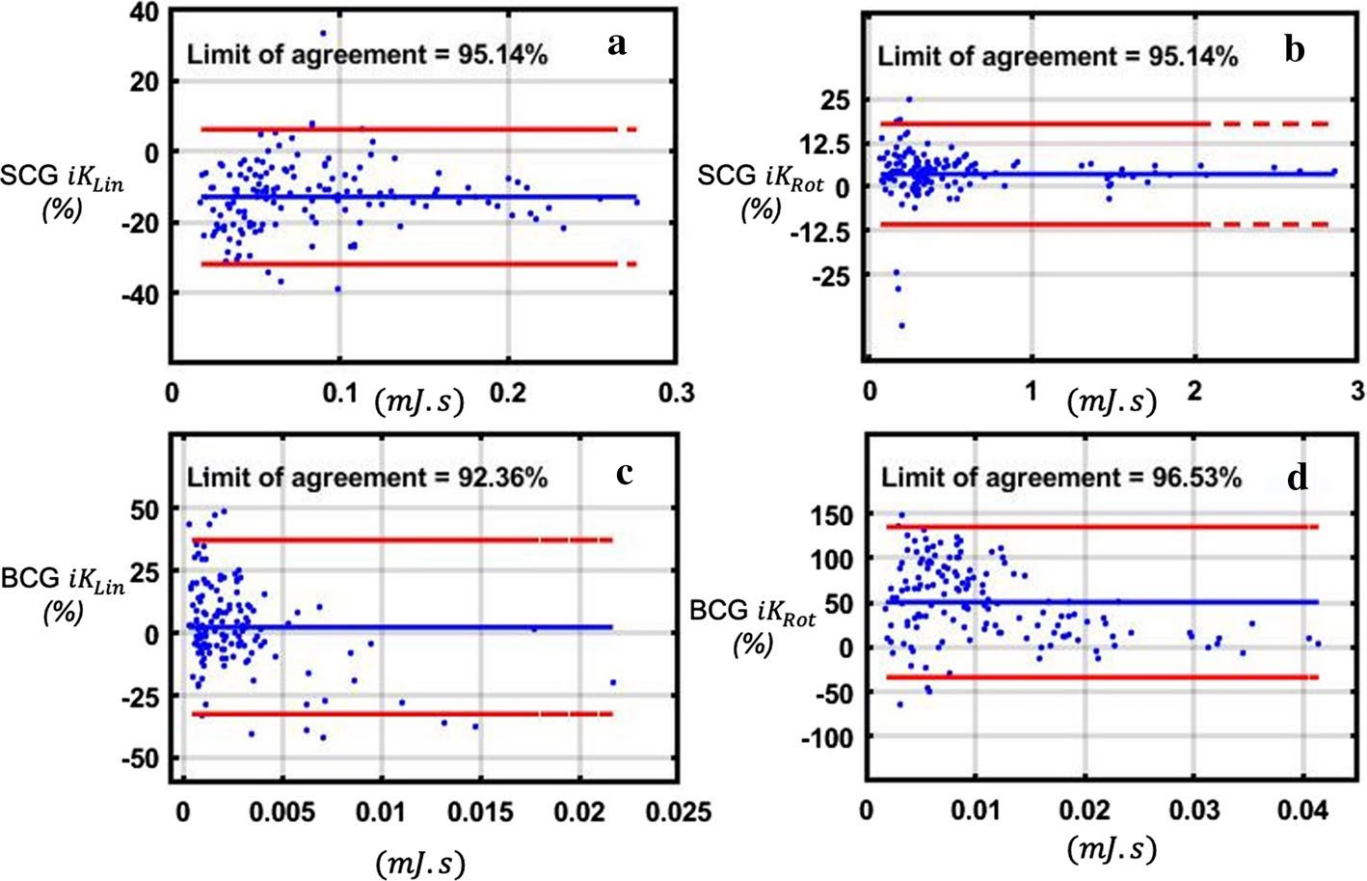
Bland–Altman plot of the KCG metrics computed from the Dev1 versus the Dev2 acquisitions. The differences (Dev2–Dev1) expressed as a percentage of the value on the axis, i.e., proportionally to the magnitude of measurements, are plotted as a function of the mean value of the two measures. **a** SCG ${{{iK}}_{{{\text{Lin}}}}}$; **b** SCG ${{{iK}}_{{{\text{Rot}}}}}$; **c** BCG ${{{iK}}_{{{\text{Lin}}}}}$; **d** BCG ${{{iK}}_{{{\text{Rot}}}}}$

The Bland–Altman plots of the KCG metrics computed from the Dev1 versus the Dev2 acquisitions are shown in Fig. 2. No trends were seen for BCG ${{iK}}_{\mathrm{Lin}}$, BCG ${{iK}}_{\mathrm{Rot}}$, SCG ${{iK}}_{\mathrm{Lin}}$, and SCG ${{iK}}_{\mathrm{Rot}}$. The limits of agreement of the compared data were 92.36% for BCG ${{iK}}_{\mathrm{Lin}}$, 96.53% for BCG ${{iK}}_{\mathrm{Rot}}$, 95.14% for SCG ${{iK}}_{\mathrm{Lin}}$, and 95.14% for SCG ${{iK}}_{\mathrm{Rot}}$. The mean difference in percentage between the two devices for each metric was 2%, 50%, 12%, and 3%, respectively (Fig. 2).

### Ensemble average length effect

**Fig. 3:**
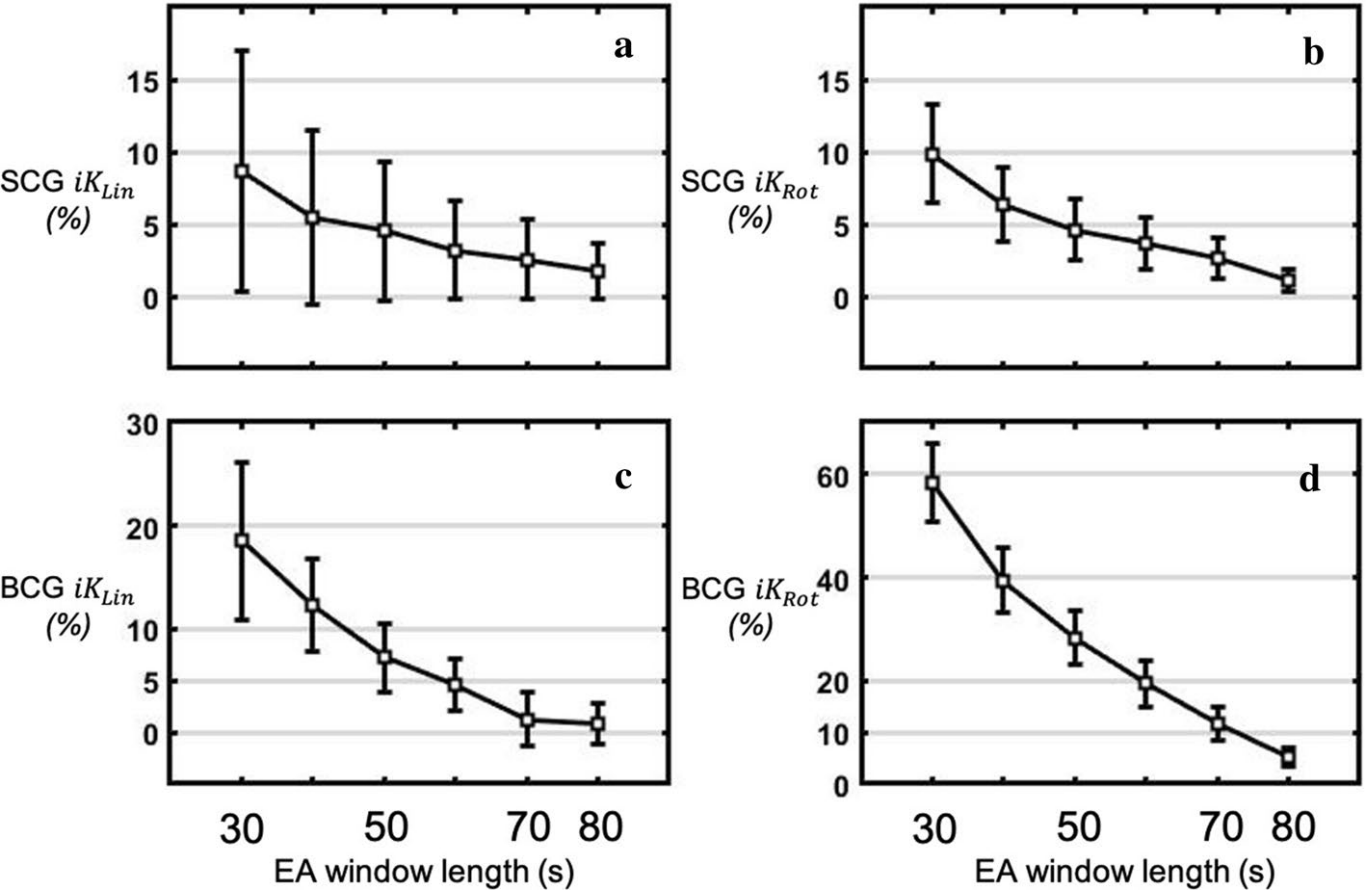
Differences expressed as a percentage of the value with 95% Confidence Interval (CI) of each KCG metric computed on several window lengths, compared to the metric computed on a 90 s window. **a** SCG ${{{iK}}_{{{\text{Lin}}}}}$; **b** SCG ${{{iK}}_{{{\text{Rot}}}}}$; **c** BCG ${{{iK}}_{{{\text{Lin}}}}}$; **d** BCG ${{{iK}}_{{{\text{Rot}}}}}$

The KCG metrics BCG ${{ iK}}_{\mathrm{Lin}}$, BCG ${{iK}}_{\mathrm{Rot}}$, SCG ${{iK}}_{\mathrm{Lin}}$, and SCG ${{iK}}_{\mathrm{Rot}}$ were computed with Dev2 based on an EA computed on increasing window lengths: the first 20 s, 30 s, 40 s, 50 s, 60 s, 70 s, 80 s, and 90 s of the record. Figure 3 shows the differences expressed as a percentage of the value with a 95% CI of each KCG metric computed on several window lengths, compared to the metric computed on a 90 s window. The SCG ${{iK}}_{\mathrm{Lin}}$ showed the fastest stabilisation, as early as 40 s of window length, the EA has a mean error inferior to 5% when compared to a 90 s window length. The SCG ${{iK}}_{\mathrm{Rot}}$ and BCG ${{iK}}_{\mathrm{Lin}}$ showed the same trends and tend to have less than 5% of error from window lengths of 50 s and a 60 s, respectively. The BCG ${{iK}}_{\mathrm{Rot}}$ showed a slower stabilisation, when computed with a 70 s window length, the metrics still had a mean error of 10% when compared to a 90 s window length. Of interest, the effect of position (supine, HDT, and HUT) on the stabilisation of the metrics showed the same trends (Fig. 6 in Appendix A).

### Position-induced haemodynamic changes

**Fig. 4:**
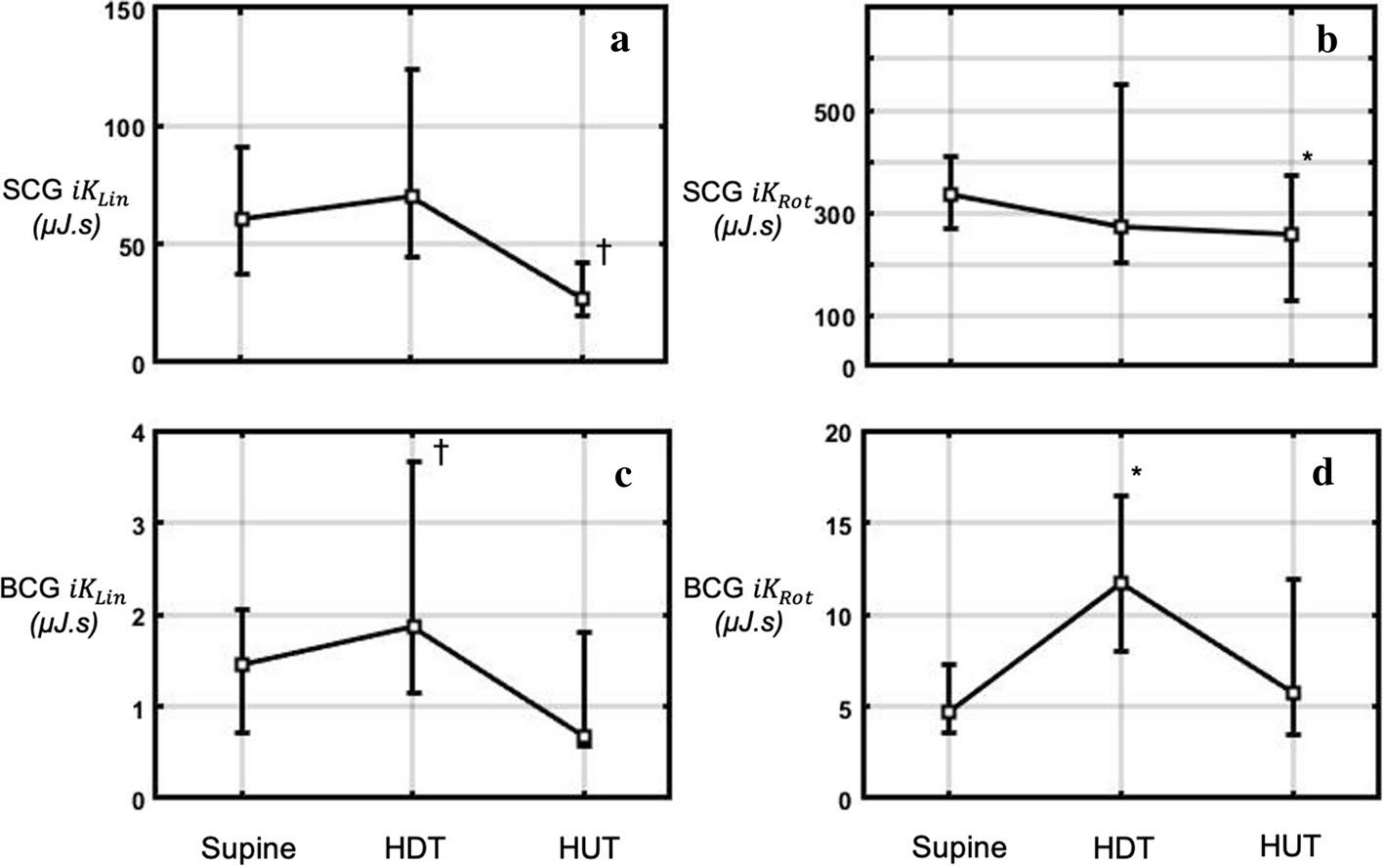
KCG metrics measured during acute change in position, from supine to HDT to HUT. **a** SCG ${{{iK}}_{{{\text{Lin}}}}}$; **b** SCG ${{{iK}}_{{{\text{Rot}}}}}$; **c** BCG ${{{iK}}_{{{\text{Lin}}}}}$; **d** BCG ${{{iK}}_{{{\text{Rot}}}}}$. Data are plotted as median [Q1; Q3] *p < 0.005 ^†^*p* < 0.0001

The linear mixed-effects model showed significant differences between positions for all KCG parameters (*p* < 0.0001). Paired *t* tests were used to compare the HDT and HUT to the supine position. The SCG ${{iK}}_{\mathrm{Lin}}$ and SCG ${{iK}}_{\mathrm{Rot}}$ were stable from the supine to the HDT positions, and decreased to values below the baseline when participants were in the HUT position (*p* < 0.0001, and *p* < 0.005, respectively). The BCG ${{iK}}_{\mathrm{Lin}}$ and BCG ${{iK}}_{\mathrm{Rot}}$ increased from the supine to HDT positions (*p* < 0.0001 and *p* < 0.005, respectively), and returned to the baseline level in the HUT position (Fig. 4).

## Discussion

### Main findings

Kinocardiography is emerging as a promising non-invasive technique to monitor cardiac inotropic activity. The main findings of this study are that the KCG metrics, namely BCG ${{iK}}_{\mathrm{Lin}}$, BCG ${{iK}}_{\mathrm{Rot}}$, SCG ${{iK}}_{\mathrm{Lin}}$, and SCG ${{iK}}_{\mathrm{Rot}}$, can be computed based on BCG and SCG signals acquired using different sensors at either 1 kHz or 50 Hz indifferently. Moreover, a recording of 60 s is enough to acquire BCG ${\mathrm{iK}}_{\mathrm{Lin}}$, SCG ${{iK}}_{\mathrm{Lin}}$, and SCG ${{iK}}_{\mathrm{Rot}}$ readings with less than 5% margin of error.

### Sampling rate effects

It has previously been shown that BCG signals’ content is expected to lay in the 0 Hz–15 Hz frequency range [12], while SCG content is expected to be in the 0 Hz–20 Hz [18], 25 Hz [19], or 30 Hz [20] frequency range. Others also argue that Phonocardiogram signals in the range superior to 25 Hz should also be considered as high-frequency seismocardiograms [21]. Both BCG and SCG have been recorded at high frequency, ranging from 200 Hz to 1 kHz [2, 12]. Acquiring these signals at a lower frequency would enable: (1) real-time transmission using low power wireless techniques, especially when multiple axes, such as the 12 used in this study, are acquired simultaneously, and (2) a reduction in the amount of data to be stored, which is a growing challenge that Internet of things (IoT) and medical devices have to tackle [22]. However, this needs to be done while maintaining the integrity and quality of the data. The scope of this work was not to evaluate if SCG and BCG were accurately acquired at 50 Hz. It was rather to evaluate if *iK* metrics computed through kinocardiography and derived from SCG and BCG signals acquired at 50 Hz were similar to the same metrics acquired at 1 kHz. In other words, to which extent SCG and BCG signals acquired at 50 Hz contain all the information needed to compute the *iK* metrics. The Bland–Altman comparison between the 1 kHz and 50 Hz acquisition showed a satisfactory limit of agreement of more than 90% for all four KCG metrics. The plots show no trends and the differences between both measures represent less than 0.1% for all four metrics. This demonstrates that the SCG and BCG *iK* metrics computed from signals recorded at 50 Hz or 1 kHz can always be considered to have a precision greater than 1‰.

### Sensor comparison

The same 4 metrics computed on the signals acquired by Dev2 at 50 Hz was comparable to the metrics computed with Dev1 acquired at 1 kHz, and demonstrated a satisfactory limit of agreement above 90% for all 4 metrics. The plots showed no trends, and the differences in the mean value of both measures were 2% for BCG ${{iK}}_{\mathrm{Lin}}$, 50% for BCG ${{iK}}_{\mathrm{Rot}}$, 12% for SCG ${{iK}}_{\mathrm{Lin}}$, and 3% for SCG ${{iK}}_{\mathrm{Rot}}$. While the limits of agreement showed that the difference of the metrics computed with Dev1 and Dev2 were constant, there was a large difference compared to the mean value of BCG ${{iK}}_{\mathrm{Rot}}$. This was likely due to the poor 100 mdps resolution of the gyroscopes used in Dev1, which was 25 times larger compared to the 4 mdps resolution of the sensors used in Dev2. This hypothesis is supported by the high-resolution gyroscopes of approximately 9 mdps used by Tadi et al. when performing gyrocardiography [23].

### Ensemble average window length effect

BCG and SCG are prone to artefacts due to non-voluntary and voluntary movements, as well as respiratory variations. Generally, an average beat is constructed using EA to mitigate these undesired noisy activities [13, 20]. However, the length of the window or the number of beats on which the EA is computed is arbitrary. Studies have applied variable average recording lengths on which the EA is computed, and these have ranged from 5 to 15 s [20], 5 to 10 heartbeats [17], and 90 s [6] to 140 s [24]. For a modified weighing scale measuring BCG, Inan et al. computed the average beat on 30 heartbeats, which is close to 30 s [25]. The EA can be computed on all the beats indistinctly or depending on the respiratory phase [24, 26] to take into account the BCG-SCG respiration variability. In this study, we showed how KCG parameters derived from BCG and SCG are modified by the length on which the EA is computed on all the heartbeats indistinctly. Our hypothesis was that after 90 s, each metric reaches a reasonably stable value, and that a longer recording would be impractical in the clinical setting. We have shown that BCG ${{iK}}_{\mathrm{Lin}}$, BCG ${{iK}}_{\mathrm{Rot}}$, SCG ${{iK}}_{\mathrm{Lin}}$, and SCG ${{iK}}_{\mathrm{Rot}}$ reach the threshold of below 10% of discrepancy after 50 s, 70 s, 30 s, and 30 s, respectively. Therefore, when using KCG metrics, depending on the amplitude of the event to be detected, if metrics are computed on different EA window lengths, attention should be given to the varying results obtained solely due to the EA window length differences. For a given metric, the IC of the mean is relatively stable, and has little dependence on the EA window length. Even with BCG ${{ iK}}_{\mathrm{Rot}}$, for which we observed a 58% mean difference between the 30 s and 90 s window lengths, the IC was consistently lower than 15% for all time frames. Hence, depending on the precision needed on the metric, short window lengths with a rather acceptable error margin could be used to compare metrics computed on the same window length.

In a previous study [6], when a small dose of dobutamine (5 µg kg^−1^ min^−1^) was administered to healthy participants, there was an increase in all the metrics; BCG ${{ iK}}_{\mathrm{Lin}}$ (50%), BCG ${{ iK}}_{\mathrm{Rot}}$ (150%), SCG ${{ iK}}_{\mathrm{Lin}}$ (100%), and SCG ${{ iK}}_{\mathrm{Rot}}$ (188%). These results help better understand the potential contribution of the EA window length in light of changes induced by haemodynamic change. Indeed, in the occurrence of dopamine-induced increases on each metric, a 30 s window length-based EA would have enabled a true positive detection of the influence of 5 gamma of dobutamine on all the KCG metrics.

During the change of position from supine to head-up tilt, HR significantly increases for all subjects. However, as one can see on Fig. 6, the dynamic of stabilisation of each metric does not change in function of the position. This result might surprise as a higher heart rate should allow more heartbeats to be included in the EA and therefore provoke a faster stabilisation of the metrics computed with the EA. However, as we speculate that EA stabilisation rate is mainly linked to respiration rate, a higher heart rate without change in respiration rate should indeed not change the stabilisation dynamic of the metrics extracted from the EA.

### HDT and HUT effects

Long term HDT-6 bed rest is used as an Earth-based analogue to the effect of microgravity exposure on the cardiovascular system [27]. While long-term—above 2 weeks—HDT-6 is known to lead to orthostatic intolerance characterised by a decrease in SV, the first 30–60 min of HDT are characterised by a significant increase in SV and central venous pressure (CVP) [28, 29]. The head-up (+ 80°) tilt is characterised by an increase in peripheral resistance, a decrease in SV, and an increase in HR [28]. The BCG ${{iK}}_{\mathrm{Lin}}$ and ${{ iK}}_{\mathrm{Rot}}$ increased significantly from the supine to the HDT positions by 52% and 51%, respectively. These results are consistent with the findings of Butler et al. on SV variation due to HDT [28] and the relationship between BCG ${{ iK}}_{\mathrm{Lin}}$ and SV [6]. However, BCG ${{ iK}}_{\mathrm{Lin}}$ and ${{ iK}}_{\mathrm{Rot}}$ did not change significantly from the supine to HUT positions, while SV is expected to decrease. In contrast, SCG ${{ iK}}_{\mathrm{Lin}}$ and ${{iK}}_{\mathrm{Rot}}$ decreased significantly from the supine to the HUT position, but did not change from the supine to the HDT position. While these results are of interest, in this study, no conclusions on the KCG ability to follow cardiovascular changes during postural changes are drawn. Indeed, the aim of the postural changes was simply to widen the range of haemodynamic activity on which the validation was performed. For a comprehensive understanding of the effects of HDT and HUT on KCG parameters, a dedicated study protocol should be performed with the presence of alternative haemodynamic measures.

### Strengths and limitations

Metrics computed with KCG are based on integration of ensemble-averaged kinetic energy signals. These metrics are automatically computed and do not necessitate the intervention of an operator. There is no need to manually select the signal window on which the computations are performed. Thus, they may be particularly suitable for real-time analysis and telemonitoring applications. Other research teams are also pursuing similar research aims. Inan et al. presented a method based on fusion of three normalised linear SCG axes and use of a graph similarity score (GSS), and found significant differences in GSS between compensated and decompensated heart failure patients [5]. Mechanical parameters extracted from KCG and GSS-derived models are more robust against motion artefacts and other disturbances, as they do not require accurate event-based detection of peaks upon which most other BCG/SCG-based methods rely. Moreover, a known issue to EA synchronised with the R-wave is its tendency to blur events that varies within a record, such as cardiac mechanical events present in the SCG or BCG signal (e.g., aortic opening, peak systolic ejection, etc.) [30]. As the method presented here is based on an integrated energy on the whole cardiac cycle, it is more robust to this kind of distortions in the waveform too.

The study presents potential limitations that require consideration. Dev1 and Dev2 sensors for BCG and SCG recordings were placed on top of each other to obtain simultaneous signals. Therefore, the sensors were not acquiring signals from the exact same position, which may have induced a difference in the accelerations and angular rate acquired between Dev1 and Dev2. Furthermore, calibration of the sensors of each device according to the manufacturer’s datasheet could have introduced a small error. We compare KCG parameters computed on signals acquired at 50 Hz and 1 kHz to highlight that the acquisition and storage of raw signals can be optimised by sampling at lower frequencies. Whereas this aim is reached here, a study searching for the optimal sampling rates for KCG parameters could be implemented in the future.

Changes in body position lead to both acute and long-term adaptations of the cardiovascular state [14]. For instance, an increase of stroke volume is reported after several minutes’ exposure to head-down tilt bed rest, because of increased venous return; however, a decrease of stroke volume is observed after several days, due to depletion of plasma volume [10]. To acquire the steadiest cardiovascular state achievable, the starting point of the records (after 10 min of rest in each position) has been chosen in such a way that acute adaptations caused by changes in body position are already established [31], while the duration of these records (up to 90 s) is significantly smaller than the time constant of the long-term adaptations (e.g., several days for adaptation of blood volume).

The maximal length of the EA window was arbitrarily chosen as 90 s. A longer window could have been used, but we opted for a recording session more suitable in a clinical setting. Notably, this was longer than the 30 s used in other studies [20, 25]. The EA was computed on all heart beats without taking the respiration phase into account. This procedure is expected to cancel the effect of respiration as it is known to have a large influence on BCG and SCG [24]. If the averaged beat was computed only on beats within a single respiratory phase (i.e., inspiration or expiration), we would expect a faster stabilisation. Also, the sub-sections of the record compared to the maximal length were always the first seconds of the record. This makes the shorter EA window length metrics less stable as they come after a transition between different respiration cycle lengths. However, these changes were determined to be small compared to the changes observed between metrics computed on different EA window lengths. Additionally, participants were subjected to a fixed respiration cycle of 10 s. We expect that a faster respiration cycle would also lead to a faster stabilisation of the metrics as the EA window would include more breathing cycles.

Given that the order of positioning from supine to HDT and to HUT was not randomised, the effect of the HDT preceding the HUT position could have impacted the measures. However, the potential effect of positioning order may have been minimised as participants were placed in the supine position for 10 min before HDT or HUT. Furthermore, the physiological effects of the changes in posture were a supplementary result of the study. Participant numbers were small, and they were all male. However, assessing 12 or fewer individuals for a validation study is common [29, 32]. The absence of female participants should not have [33], or may only slightly have affected [31] the conclusions drawn from the postural changes. It has been shown that the BCG and SCG waveforms are affected by gender [34], and these differences are hypothesised to be mainly due to the inherent cardiac physiology distinction that can be found between men and women [35]. Therefore, the relatively wide haemodynamic range induced by head tilts in this study allows to partially mitigate the absence of females. However, a validation performed on a larger group including females would be interesting as a next step to confirm our findings. Also, these validations were performed on a relatively young participants group, as it is common to see on validation studies [29, 33]. Despite the points highlighted here, we believe that these potential limitations do not preclude the conclusions brought by this research.

## Conclusions

In this study, time integrals of kinetic energy (*iK*), based on the ensemble average of Ballistocardiography and Seismocardiography signals, were computed on a relatively wide range of haemodynamic statuses induced by the acute head-up and head-down tilt positions. The *iK* were computed simultaneously using two different sensors, at high frequency (1 kHz) and lower frequency (50 Hz), and at an increasing duration of recording time. These metrics showed to be robust in light of sensor differences, sufficiently accurate when BCG and SCG were acquired at 50 Hz, and accurate with a precision of ± 5% for most of the tested metrics when computed on a 60 s record length. These results support the use of kinocardiography to monitor cardiac inotropic activity non-invasively and robustly complement information obtained using standard techniques in clinical settings.

## Methods

### Protocols and participants

Twelve healthy male volunteers who had no history of cardiac disease were non-smokers, and had BMIs between 20 and 25 kg m^−2^, were recruited. Participants had a mean age of 22.1 years (± 3.1), and a mean BMI of 23.2 kg m^−2^ (± 4.2). None of the participants took drugs or any medication. The study protocol complied with the Declaration of Helsinki, was approved by the local Ethics Committee (Hôpital Erasme—CCB: B406201630013), and was registered on ClinicalTrials.org (April 11, 2017), identifier *NCT03107351*. The prototype of the KCG device used in this clinical trial was authorised by the Belgian Federal Agency for Medicine and Health Products (FAMHP). Written informed consent was obtained from each participant prior to the experimental testing procedure.

Sensors were placed on participants to connect to the ECG, BCG, and SCG recording systems. They had to lie in a supine position on an inversion table for 10 min for stabilisation. The recording was then started, and continuous data were acquired for the next 7 min. Participants were then put in HDT of 6 degrees for 10 min of stabilisation followed by 7 min of recording. In the final position in HUT of 80 degrees, the participants underwent another 10 min of stabilisation, followed by 7 min of recording.

Participants were asked to remain as still as possible, and to not talk or fall asleep for the duration of the recording.

The influence of breathing on the BCG signal is well known [3, 24, 36]. Hence, we accounted for the influence of respiration using an Imposed and Controlled Breathing (ICB) protocol. The ICB consisted of breathing for a fixed duration during the inspiration and expiration phases matching the normal inspiratory over total breath ratio (35%). An audio system with inspiratory and expiratory sounds was used to guide the participants. Participants were briefed with breathing instructions prior to performing 10 repetitions of 4-, 6-, 8-, and 10-s breathing cycles in succession. Post-hoc analysis showed that they maintained these cycles accurately.

### Data acquisition and devices

Two devices (Dev1 and Dev2) were used to simultaneously record the ECG, SCG, and BCG signals of the participants. Dev1 is the Cardiovector (Medical Computer Systems Ltd., Zelenograd, Russian Federation), a system used to monitor autonomic function on the International Space Station [37]. It allows the synchronous performance of: (a) electrocardiography (ECG), (b) impedance cardiography (ICG) in tetrapolar configuration, (c) plethysmography (PTG) using a nasal thermistor to evaluate breathing, (d) 6-DOF SCG (3-axis linear accelerations and 3-axis angular velocities, LIS344ALH and LPY403AL, STMicroelectronics), and (e) 6-DOF BCG (3-axis linear accelerations and 3-axis angular velocities, LIS344ALH and LPY403AL, STMicroelectronics). The acceleration was acquired at 0.5 mg and an RMS noise of 500 μg·Hz^−1/2^, while the angular rate was acquired at a 100 mdps resolution and 10 mdps·Hz^−1/2^ RMS noise. An 80 Hz first-order analogue low-pass filter was applied to both linear accelerations and angular rates. All signals were sampled at 1 kHz and stored on a computer. The Dev1 had separate small plastic covers containing the accelerometer and gyroscope sensors that could be individually attached to the body. More details on the system can be found in [37].

Dev2 was the kinocardiograph (HeartKinetics SRL, Gosselies, Belgium), a portable device with two modules. Each module contained a 3-axis accelerometer and 3-axis gyroscope sensor (LSM6DSL, STMicroelectronics), and was attached to the body with standard sticky gel ECG electrodes (3 M). The acceleration and angular rate sensitivity range of the sensor were set to ± 2 g and ± 250 dps, respectively, with a resolution of 0.061 mg/LSB and 4.375 mdps/LSB and an RMS noise of 80 μg·Hz^−1/2^ and 4 $\mathrm{mdps}\cdot{Hz}^{-1/2}$ with an output bandwidth of 416 Hz. The Dev2 was controlled with a smartphone or a tablet connected via Bluetooth. It collects a two-lead ECG at 200 Hz (ADS1292R, AD Instruments) together with 3-DOF linear accelerations and 3-DOF rotational velocities from each detector. A micro-controller (STM32F411, STMicroelectronics) was used to acquire synchronously data from each chip and operate the Bluetooth low energy (SPTLE-RF, STMicroelectronics). A total of 12-DOF linear acceleration and angular velocity signals were recorded at 50 Hz, and a 25 Hz first-order analogue low-pass filter was applied.

**Fig. 5:**
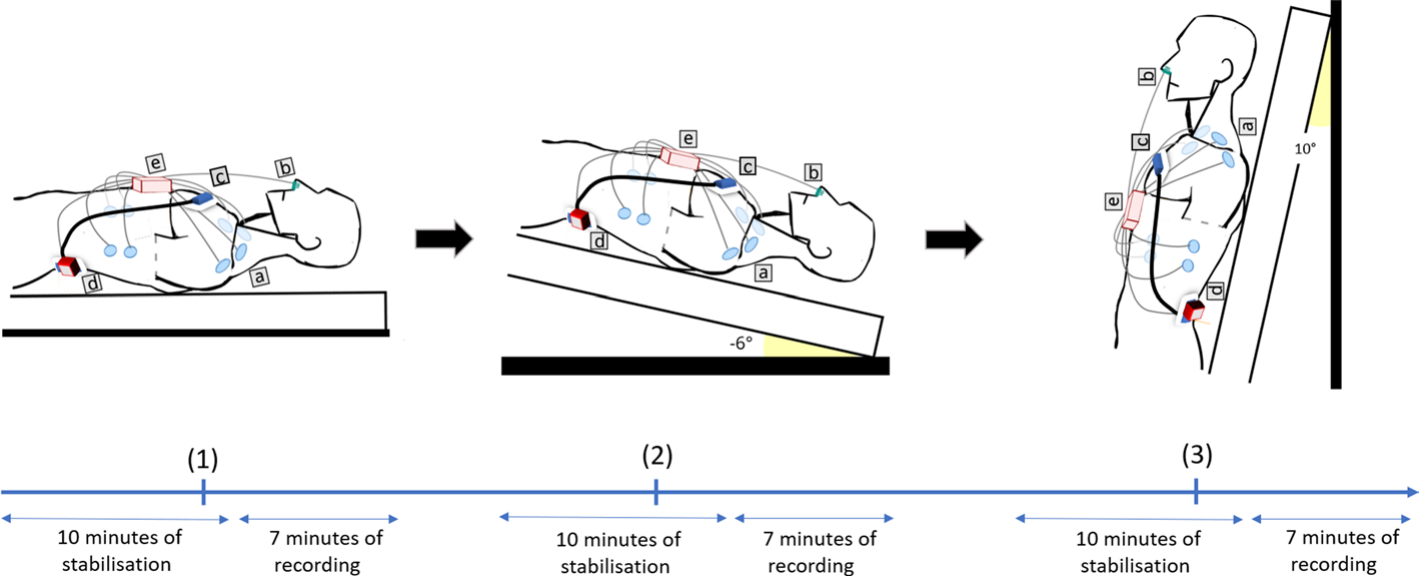
Experimental setup of different elements of Dev1 and Dev2 in 3 positions: (1) Supine, (2) HDT, and (3) HUT. **a** Dev1 ECG/ICG electrodes; **b** Dev1 PTG sensor (nasal thermistor); **c** Dev1 & Dev2 SCG sensors secured together on the sternum; **d** Dev1 & Dev2 BCG sensors secured together in the lumbar lordosis curvature; **e** Dev1 main unit (connection and amplification)

**Fig. 6:**
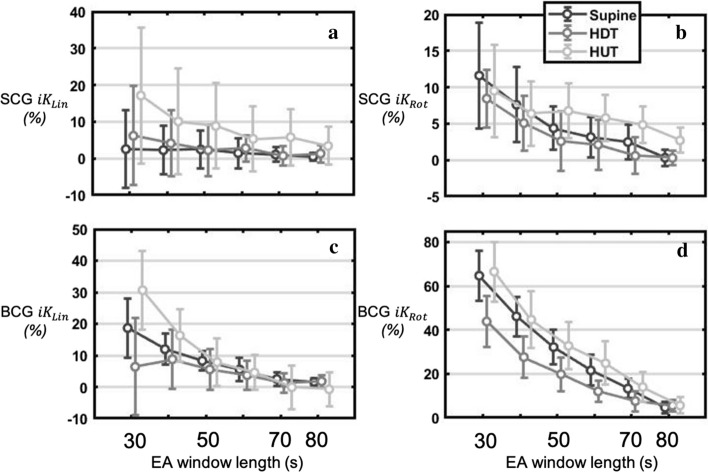
Differences expressed as a percentage of the value with 95% Confidence Interval (CI) of each KCG metric computed on several window length compared to the metric computed on a 90 s window for each position. **a** SCG ${{{iK}}_{{{\text{Lin}}}}}$; **b** SCG ${{{iK}}_{{{\text{Rot}}}}}$; **c** BCG ${{{iK}}_{{{\text{Rot}}}}}$; **d** BCG ${{{iK}}_{{{\text{Rot}}}}}$

Dev2 consists of two parts which corresponds to the SCG and BCG sensors. For both BCG and SCG, the Dev1 sensors were adhesively attached on top of the Dev2 sensors, which were then positioned on the body with their ECG electrodes. The BCG sensors were placed in the lumbar lordosis curvature, between the second and the third lumbar vertebrae and close to the participant’s centre of mass. The SCG sensors were placed on the manubrium of the sternum, below the clavicle, and in the superior mediastinum region where the great vessels emerge from the heart (Fig. 5).

Data acquisition from both the Dev1 and Dev2 devices was initiated at the same time, and continuous data were acquired for the duration of the recording.

### Data analyses

All data analyses and statistics were performed on a custom-built KCG analysis toolbox written in Matlab 2018b (Mathworks, NL).

Signals acquired using Dev1 and Dev 2 were synchronised based on the ECG signal. The delay between the recordings of the two devices was determined by cross-correlating the ECG signals from both devices to find the point of correspondence. Signal synchronisation was validated visually for all records. The EA method, which is described in the next section, is based on the R waves detected on the ECG. However, there may have been slight differences in the ECG readings of the two devices and in the synchronisation of the ECG of both Dev1 and Dev2. Therefore, the ECG of Dev1 was used for the rest of the analyses for both devices.

The SCG and BCG signals of Dev2 were up-sampled to 1 kHz. Detection of the P, Q, R, S, and T waves was conducted using a modified Pan and Tompkins algorithm [38], and a pattern matching algorithm on the ECG channel. The detections were validated visually. The heart rate (HR) was calculated based on the RR intervals. Afterwards, the initial record was divided into 4 sub-records, one for each of the 4 phases of the ICB protocol (4-, 6-, 8-, and 10-s breathing cycles), and each sub-record was processed independently. In this study, only data from the 10-s breathing cycle protocol are presented. This was done to obtain the longest record possible within a constant breathing cycle.

#### Ensemble average method

The time reference for the EA is a fixed interval that took into account the cardiac activity preceding atrial depolarisation occurring before the R peak. Indeed, the beginning of the *n*th interval started before the P wave, at time ${k}_{n,\mathrm{start}}= {R}_{n}- \Delta$, where ${R}_{n}$ is the time of the *n*-th R peak and ∆ is 200 ms. The end of the cardiac cycle of interest was assumed to be ${k}_{n,\mathrm{end}}= {R}_{n}+ \mathrm{max}[{RR}_{i}]$ where ${RR}_{i}$ represent all the RR intervals of the current record. The mean was taken on all the beats to obtain an average ECG signal. Based on this, an EA was calculated for each channel of the BCG and SCG recordings.

#### Kinocardiography data analysis

Participant height and weight were used to assess their inertial parameters [39]. Linear accelerations ($\vec{a}$) first undergo single-time integration to provide velocity ($\vec{v}$). From this, the linear and rotational kinetic energy transmitted by cardiac contraction to the body was computed using the following equations on the ensemble-averaged signals:

1 $$K_{{{\text{Lin}}}} = \frac{1}{2}m(v_{x}^{2} + v_{y}^{2} + v_{z}^{2} )$$

2 $$K_{{{\text{Rot}}}} = \frac{1}{2}\left( {I_{xx} \omega_{x}^{2} + I_{yy} \omega_{y}^{2} + I_{zz} \omega_{z}^{2} } \right),$$

where $m$ is the body mass of the subject, $K_{{{\text{Lin}}}}$ is the linear kinetic energy, $v_{x}$, $v_{y}$, $v_{z}$ are components of the velocity vector $\vec{v}$, $K_{{{\text{Rot}}}}$ is the rotational kinetic energy, $I_{xx}$, $I_{yy}$,${\text{ and }}I_{zz}$ are the orthogonal components of the moment of inertia, and $\omega_{x}$, $\omega_{y} ,$ and $\omega_{z}$ are components of the measured angular velocity $\vec{\omega }$ coming from the gyroscope.

These computations gave the following ensemble-averaged signals: BCG $K_{{{\text{Lin}}}}$, BCG $K_{{{\text{Rot}}}}$, SCG $K_{{{\text{Lin}}}}$, and SCG $K_{{{\text{Rot}}}}$. The time integral (*iK*) of *K* over the ensemble-averaged cardiac cycle (CC) was computed as follows:

3 $${\text{SCG}} \;{{\text{iK}}_{{{\text{Lin}}}}} = \mathop \int \limits_{{{\text{CC}}}}^{{}} {\text{SCG }}K_{{{\text{Lin}}}} (t) {\text{d}}t$$

4 $${\text{SCG iK}}_{{{\text{Rot}}}} = \mathop \int \limits_{{{\text{CC}}}}^{{}} {\text{SCG }}K_{{{\text{Rot}}}} (t){\text{ d}}t$$

5 $${\text{BCG iK}}_{{{\text{Lin}}}} = \mathop \int \limits_{{{\text{CC}}}}^{{}} {\text{BCG }}K_{{{\text{Lin}}}} (t){\text{d}}t$$

6 $${\text{BCG iK}}_{{{\text{Rot}}}} = \mathop \int \limits_{{{\text{CC}}}}^{{}} {\text{BCG K}}_{{{\text{Rot}}}} (t){\text{d}}t.$$

Where CC, which denotes the entire cardiac cycle, defined as starting with the P wave of the cycle i and ending with the P wave of the cycle *i* + 1, was delimited based on the ensemble-averaged ECG. The integral was indeed performed on this specific CC interval and not the whole ensemble-averaged signal. This leads to the scalar metrics BCG ${{{iK}}_{{{\text{Lin}}}}} ,$ BCG ${{{iK}}_{{{\text{Rot}}}}}$, SCG ${{{iK}}_{{{\text{Lin}}}}}$, and SCG ${{{iK}}_{{{\text{Rot}}}}}$.

Detailed information on the signal processing of multidimensional BCG and SCG records can be found in our previous study [6].

### Statistical analyses

#### Influence of sample rate and sensor comparison

Based on the 1 kHz acquisitions of Dev1, the sample rates BCG and SCG data were decreased by a factor of 20 by keeping the first sample and then every 20th sample after the first. Signals at 50 Hz were therefore obtained. Bland-Altmann plots [40] were generated for each KCG metric calculated on the initial 1 kHz acquisition versus the down-sampled version at 50 Hz acquisition. To compare KCG metrics computed on basis of SCG and BCG signals acquired by different sensors, Bland–Altmann plots were also generated for each metric to compare Dev1 and Dev2 acquisitions. The measures were considered similar when Bland–Altmann plots displayed (1) an absence of positive or negative trend and (2) the presence of more than 90% of the points between ± 1.96 standard deviation (STD).

#### Ensemble average window length effect

Based on the 100 s signal from the 10 repetitions of 10 s breathing cycle, each record was categorised into 8 sections which corresponded to the first 20 s, 30 s, 40 s, 50 s, 60 s, 70 s, 80 s, and 90 s of the signal, respectively, to account for the influence of duration on the EA metrics. Data were grouped according to the different positions (supine, HDT, and HUT). To quantify the difference in the EA metrics caused by the window lengths, a plot of the differences expressed as a percentage of the value [(Method A − Method B)/mean %)] with 95% Confidence Interval (CI), as described by Giavarina [40], was generated.

#### Influence of position

The KCG parameters were entered into a linear mixed-effects model [41] together with position and time as fixed effects to assess their interactions. The random effects were the intercepts for the subjects. Indeed, position and time are considered as fixed effects,, because their impact on haemodynamics is expected to be seen on the variables of interest by a change of the intercept. This fixed effect on the intercept is subject to some randomness caused by inter-individual differences, hence the choice to consider subjects as a random effect. Visual inspection of the residual plots was performed to detect any deviation from homoscedasticity or normality. *p* values were obtained using likelihood ratio tests of the full model with the effect in question, against the model without this effect. Statistical significance was set at α = 0.05 (two-tailed hypothesis tests). KCG parameters were compared between positions by a paired *t* test for data with a normal distribution, or a Wilcoxon signed-rank test for skewed data. A Lilliefors test was used to test whether the difference between the compared sample populations was normally distributed. A Bonferroni correction was applied to account for multiple comparisons. Therefore, as 4 metrics were computed, a *p* value less than 0.0125 was considered to compute 95% confidence intervals.

## Figures and Tables

**Fig. 7:**
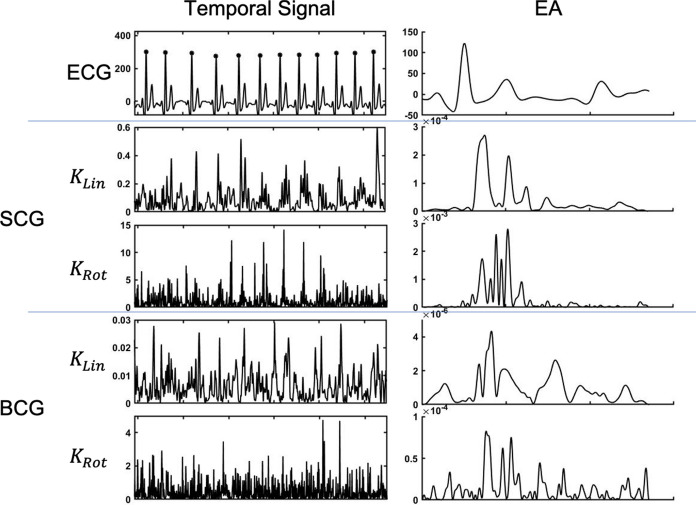
Left column are temporal signal while right column are the Ensemble Average (EA) of its corresponding signal. From top to bottom: ECG, SCG $K_{{{\text{Lin}}}}$, SCG $K_{{{\text{Rot}}}}$, BCG $K_{{{\text{Lin}}}}$, and BCG $K_{{{\text{Rot}}}}$

## Data Availability

The datasets used and/or analysed during the current study are available from the corresponding author on reasonable request.

## Appendix A

The KCG metrics BCG ${{iK}}_{\mathrm{Lin}}$, BCG ${{iK}}_{\mathrm{Rot}}$, SCG ${{iK}}_{\mathrm{Lin}}$, and SCG ${{iK}}_{\mathrm{Rot}}$ were computed with Dev2 based on an EA computed for increasing windows lengths, using the first 20, 30, … and 90 s of the record. Figure 6 shows differences expressed as a percentage of the value with 95% CI for each KCG metric computed on several window lengths compared to the metric computed on a 90 s window. The HDT position shows the fastest stabilisation, while HUT seems to delay the stabilisation of the metrics as a function of the window length.

## Appendix B

Figure 7 shows linear and rotational kinetic energy for BCG and SCG signals for one subject in HDT position.
